# VRK1 Regulates Sensitivity to Oxidative Stress by Altering Histone Epigenetic Modifications and the Nuclear Phosphoproteome in Tumor Cells

**DOI:** 10.3390/ijms25094874

**Published:** 2024-04-30

**Authors:** Elena Navarro-Carrasco, Eva Monte-Serrano, Aurora Campos-Díaz, Frank Rolfs, Richard de Goeij-de Haas, Thang V. Pham, Sander R. Piersma, Paula González-Alonso, Connie R. Jiménez, Pedro A. Lazo

**Affiliations:** 1 Instituto de Biología Molecular y Celular del Cáncer, Consejo Superior de Investigaciones Científicas (CSIC), Universidad de Salamanca, E-37007 Salamanca, Spain; elena.navarro@usal.es (E.N.-C.); evamonte26@usal.es (E.M.-S.); aucamposd@usal.es (A.C.-D.); paula.ga@usal.es (P.G.-A.); 2 Instituto de Investigación Biomédica de Salamanca (IBSAL), Hospital Universitario de Salamanca, E-37007 Salamanca, Spain; 3 OncoProteomics Laboratory, Cancer Center Amsterdam, Amsterdam UMC, Vrije Universiteit, 1081 HV Amsterdam, The Netherlands; f.rolfs@amsterdamumc.nl (F.R.); r.dehaas@amsterdamumc.nl (R.d.G.-d.H.); t.pham@amsterdamumc.nl (T.V.P.); s.piersma@amsterdamumc.nl (S.R.P.); c.jimenez@amsterdamumc.nl (C.R.J.)

**Keywords:** VRK1, oxidative stress, histone, acetylation, methylation, chromatin, nuclear phosphoproteins

## Abstract

The chromatin organization and its dynamic remodeling determine its accessibility and sensitivity to DNA damage oxidative stress, the main source of endogenous DNA damage. We studied the role of the VRK1 chromatin kinase in the response to oxidative stress. which alters the nuclear pattern of histone epigenetic modifications and phosphoproteome pathways. The early effect of oxidative stress on chromatin was studied by determining the levels of 8-oxoG lesions and the alteration of the epigenetic modification of histones. Oxidative stress caused an accumulation of 8-oxoG DNA lesions that were increased by VRK1 depletion, causing a significant accumulation of DNA strand breaks detected by labeling free 3′-DNA ends. In addition, oxidative stress altered the pattern of chromatin epigenetic marks and the nuclear phosphoproteome pathways that were impaired by VRK1 depletion. Oxidative stress induced the acetylation of H4K16ac and H3K9 and the loss of H3K4me3. The depletion of VRK1 altered all these modifications induced by oxidative stress and resulted in losses of H4K16ac and H3K9ac and increases in the H3K9me3 and H3K4me3 levels. All these changes were induced by the oxidative stress in the epigenetic pattern of histones and impaired by VRK1 depletion, indicating that VRK1 plays a major role in the functional reorganization of chromatin in the response to oxidative stress. The analysis of the nuclear phosphoproteome in response to oxidative stress detected an enrichment of the phosphorylated proteins associated with the chromosome organization and chromatin remodeling pathways, which were significantly decreased by VRK1 depletion. VRK1 depletion alters the histone epigenetic pattern and nuclear phosphoproteome pathways in response to oxidative stress. The enzymes performing post-translational epigenetic modifications are potential targets in synthetic lethality strategies for cancer therapies.

## 1. Introduction

The chromatin structure is highly organized in cells, and the degree of chromatin compaction determines the accessibility of DNA to reactive oxygen species, the main source of endogenous DNA damage, and to treatments based on DNA damage [1,2]. The proteins regulating chromatin dynamic organization are likely to determine the sensitivity to DNA damage-based cancer treatments. Nucleosomes are dynamic structures composed of histones that have different patterns of epigenetic modifications depending on the cellular requirements [3]. Thus, any change that affects the chromatin organization, such as the pattern of histone post-translational modifications (PTMs), can have a major effect on the chromatin remodeling associated with specific functions. Among them, there is increasing evidence indicating that chromatin changes influence the response to oxidative stress [4]. The coordination of different histone PTMs underlies the dynamic remodeling of chromatin in different biological processes. Oxidative stress drastically alters histone PTMs as well as the state of chromatin-modifier enzymes [2]. Therefore, alterations in the regulation of chromatin remodeling associated with the response to oxidative stress can contribute to facilitating the progression of human diseases, including cancer, neurological diseases, and aging [2,4,5].

Oxidative stress is the main source of endogenous DNA damage [6,7] and also the mechanism of DNA damage by ionizing radiation that generates reactive oxygen species. Oxygen reacts with guanine residues, forming 8-oxoguanine (8-oxoG) [8], a DNA lesion that is very efficiently removed by 8-oxoguanine DNA glycosylase (OGG1) [9]. However, unrepaired 8-oxoG lesions can lead to single-strand DNA breaks that progress to double-strand breaks [10]. The local histone environment of chromatin mediates the recruitment of OGG1 [11,12], which is influenced by the nucleosome wrapping [13]. Therefore, alterations in the chromatin organization are likely to alter the DNA repair. DNA oxidative lesions contribute to neuronal DNA damage, which is associated with neurological diseases [14]. The excess of reactive oxygen species (ROS) in the microenvironment of normal cells facilitates genome instability [15] and tumorigenesis [16]. Many tumors have elevated levels of ROS and chronic oxidative stress [17,18]. Chromatin remodeling is associated with tumor cell adaptation to chronic oxidative stress [4,19]. Moreover, cancer treatments such as radiotherapy take advantage of this ROS excess in tumor cells by causing an accumulation of lethal levels of ROS and, hence, increasing oxidative DNA damage to induce tumor cell death [20]. In the context of cancer, oxygen plays a critical role in the response to some local treatments, such as ionizing the radiation that indirectly alters DNA by generating reactive oxygen species [4,8]. Oxidative DNA lesions locally alter the chromatin organization, which is conditioned by the pattern of the epigenetic modifications of histones [21]. Both the prevention of ROS accumulation and the repair of the DNA damage caused by ROS are crucial to preserve genome integrity and cellular viability [4].

The alteration of nucleotides by oxidative stress can affect the pattern of epigenetic modifications of histones as part of the DNA damage response (DDR), a process requiring dynamic chromatin remodeling to facilitate its different sequential steps [22,23,24]. Chromatin structural modifications have several roles in DDR, like the induction of chromatin relaxation, which is essential to initiate the repair response. Moreover, these changes act as sensors, amplifying the signal and functioning as scaffolds for the recruitment of downstream signaling and sequential repair proteins, which vary depending on the type of DNA damage. Changes in the chromatin structure vary depending on the location and the type of lesion, thus initiating different cell-cycle checkpoints and repair pathways for specific cellular requirements [25]. Alterations in the chromatin dynamic reorganization can impair the DNA repair mechanisms. In response to DNA damage, the acetylation of histones facilitates local chromatin relaxation and the accessibility to DDR proteins. Tip60 acetylates H4 in K16, which impairs the interaction between the H4 tail and adjacent nucleosomes, increasing the chromatin accessibility to the repair machinery [26], in addition to key DDR proteins such as ATM, which is activated by VRK1 [27] and is linked to the chromatin relaxation associated with H4K16ac [28]. These acetylations are triggered by VRK1, which specifically phosphorylates Tip60/KAT5, leading to the activation of its transacetylase activity [29,30], which acetylates ATM, a modification required for this kinase activation [31,32]. VRK1 also regulates the pattern of histone epigenetic modifications [33], which can interfere with the DNA damage responses. Alterations in the regulation of histone epigenetic modifications are associated with a defective DDR and have been linked to human pathologies like cancer [34,35]. DDR deficiencies due to changes in the chromatin structure result in genome instability and mutagenesis [36], such as the loss of function of SETD2, the methyltransferase that carries out the H3K36me3 post-translational modification (PTM), which promotes renal cancer progression due to DDR impairment [37]. In response to oxidative stress, the post-translational modifications in histone H3 are modulated through the regulation of epigenetic enzymes, such as the reduction in HDAC activity [38]. ROS also regulates histone methylation, including activating PTMs (H3K4me2 and H3K4me3) and repressive PTMs (H3K9me2, H3K9me3, H3K27me2, and H3K27me3), which vary depending on the cellular requirements [2,39].

VRK1 is a serine–threonine kinase that, during evolution, appeared late in complex organisms, probably to coordinate and integrate many basic cellular functions in parallel to p53 [40,41]. In *Drosophila melanogaster*, this gene (*ballchen*) has been renamed to nucleosomal-kinase 1 (NHK-1), which better reflects its cellular functions [42,43,44,45,46,47]. VRK1 is a nuclear and chromatin kinase that interacts with nucleosomes [48] and participates in chromatin remodeling as an epigenetic writer by directly phosphorylating histones in response to stimuli [49,50], or indirectly by regulating the epigenetic enzymes that perform histone post-translational modifications (PTMs) [29,30,51]. VRK1 depletion by itself alters the pattern of histone H3 acetylation and methylation in several lysine residues [33]. VRK1 binds to and phosphorylates histone H3 at Thr3 and Ser10, which, together with Aurora B, participates in the sequential steps of chromatin condensation during mitotic progression.

VRK1 regulates the nuclear and chromatin phosphoproteome and affects different signaling pathways implicated in chromatin remodeling [52]. VRK1 participates in the initiation of the DDR, promoting chromatin remodeling by the direct phosphorylation of Tip60/KAT5 [29,30], H3 [48] H2A [49], and H2AX [53], which are associated with local chromatin relaxation and facilitate the access to DNA damage of the sensing and repair mechanisms. In addition, VRK1 also phosphorylates and regulates sequential DNA repair proteins from DNA damage repair (DDR) pathways such as NBS1, 53BP1, and p53 [53]. VRK1 malfunction or downregulation leads to genome instability and DNA damage accumulation because of a defective DDR [53]. Moreover, VRK1 is associated with the regulation of chromatin remodelers such as the direct phosphorylation and activation of the acetyltransferase Tip60/KAT5 in response to doxorubicin-induced DNA damage [29,30]. These data indicate a potential function of VRK1 in chromatin remodeling that could influence the oxidative stress response, and histone phosphorylations are likely to alter the pattern of their epigenetic modifications. Moreover, VRK1 overexpression is associated with a poor prognosis in several tumor types [54,55]. The VRK1 roles vary depending on the phase of the cell cycle; in some contexts, it promotes tumor growth and in others tumor prevention [53]. An additional pathogenic implication of the role of VRK1 is associated with rare variants of VRK1, which are functionally defective. These rare VRK1 variants are functionally involved in very severe motor neuron diseases, among which are spinal muscular atrophy, amyotrophic lateral sclerosis, and distal hereditary neurological diseases [56].

In this work, we have studied the implications of VRK1 on the cellular sensitivity to oxidative stress and its role in the modification of histone post-translational modifications and the nuclear phosphoproteome, which underly the sensitization of tumor cells to treatments based on DNA damage.

## 2. Results

### 2.1. VRK1 Depletion Causes an Increase in 8-Oxo-Guanine DNA Lesions Induced by Oxidative Stress

**Figure 1:**
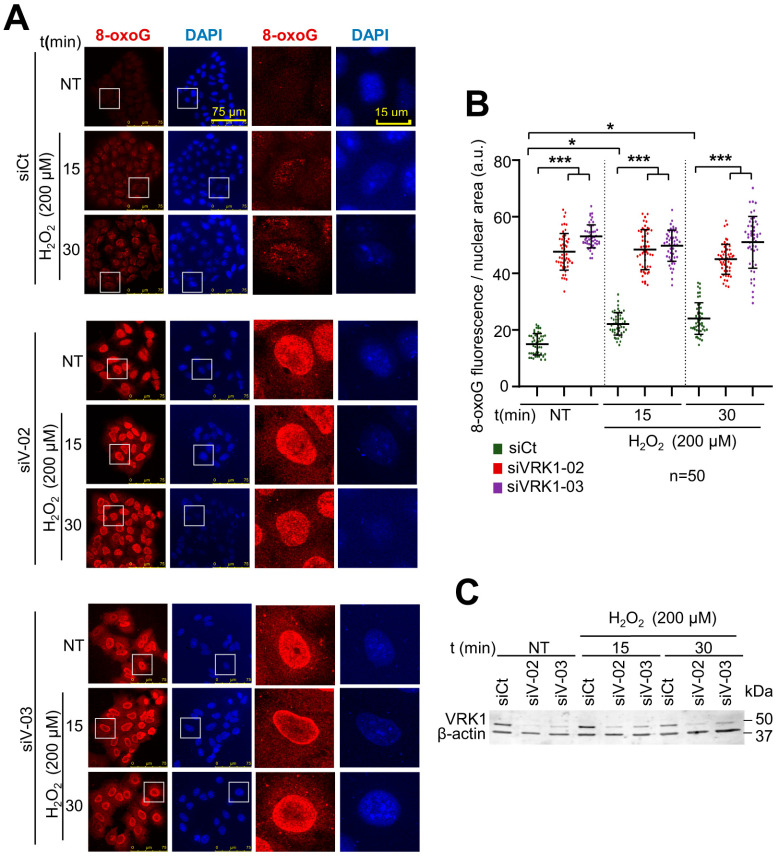
Accumulation of 8-oxoG DNA lesions in serum-deprived A549 lung adenocarcinoma cells treated with hydrogen peroxide at the indicated times. (**A**) Immunofluorescence of A549 cells treated with hydrogen peroxide at different time points in control (siCt) or VRK1-depleted cells stained by IF and detected using confocal microscopy. The boxed cells are individually shown on the right. (**B**) Quantification of the levels of 8-oxoG induced by hydrogen peroxide in the experiment shown in part a. VRK1 depletion with two different siVRK1s enhances significantly by itself the accumulation of 8-oxoG lesions in DNA. (**C**) Immunoblot showing VRK1 levels. β-actin was used as a loading control. * *p* < 0.05; *** *p* < 0.001; a.u.: arbitrary units; siCt: siControl; siV-02: siVRK1-02; siV-03: siVRK1-03. NT: non-treated. Experiments were independently performed three times.

Oxidative damage causes 8-oxoG lesions in the DNA [57], which locally alter chromatin [4,19]. VRK1 depletion also alters the pattern of epigenetic histone modifications, which impair chromatin dynamics and accessibility [29,33,51]. Therefore, to analyze the possible effect of VRK1 knockdown on the oxidative stress response, we studied the accumulation of oxidative DNA damage in the absence of this kinase in cells treated with hydrogen peroxide. VRK1 depletion is required for exiting G0 in the cell cycle. Thus, we used cell cycle-arrested cells by serum deprivation [58]. For this aim, we determined whether VRK1 depletion could affect the level of DNA oxidation, which was detected by 8-oxoG lesions. VRK1 was independently depleted with two different siRNAs (siVRK1-02 and siVRK1-03) in A549 lung adenocarcinoma (Figure 1) cells, followed by serum deprivation (0.5% FBS) for 48 h and treated with 200 µM H_2_O_2_ for 15 and 30 min to accumulate cells in G0 and avoid the mitogenic signals that activate VRK1 and induce cell cycle progression [58,59]. In these cells, the 8-oxoG DNA lesions were detected by immunofluorescence. In control cells, the level of 8-oxoG was low and the accumulation of 8-oxoG induced by hydrogen peroxide was significant but relatively low (Figure 1A, siCt panel; Figure 1B, green). However, VRK1 depletion by itself facilitated the accumulation of 8-oxoG even in non-treated cells (NT), which indicated that the loss of VRK1 by itself sensitized the DNA to the endogenous level of reactive oxygen species before treatment (Figure 1A, NT lanes; Figure 1B, red and purple), and, because the level of 8-oxoG lesions was very high, the additional treatment with hydrogen peroxide had a minor additional effect (Figure 1A, center and lower panel). This effect of VRK1 depletion on the accumulation of 8-oxoG DNA lesions is independent of the presence of serum (Supplementary Figure S1) and is a consequence of the impairment of chromatin dynamics required for DNA repair [11,12,13]. VRK1 depletion caused a similar effect on the 8-oxoG levels in LN229 glioblastoma cells exposed to hydrogen peroxide, but the oxidative damage response was slower and detected at a later time (Supplementary Figure S2).

### 2.2. VRK1 Depletion Facilitates the Accumulation of Free 3′-Ends in the DNA Induced by Oxidative Stress

**Figure 2:**
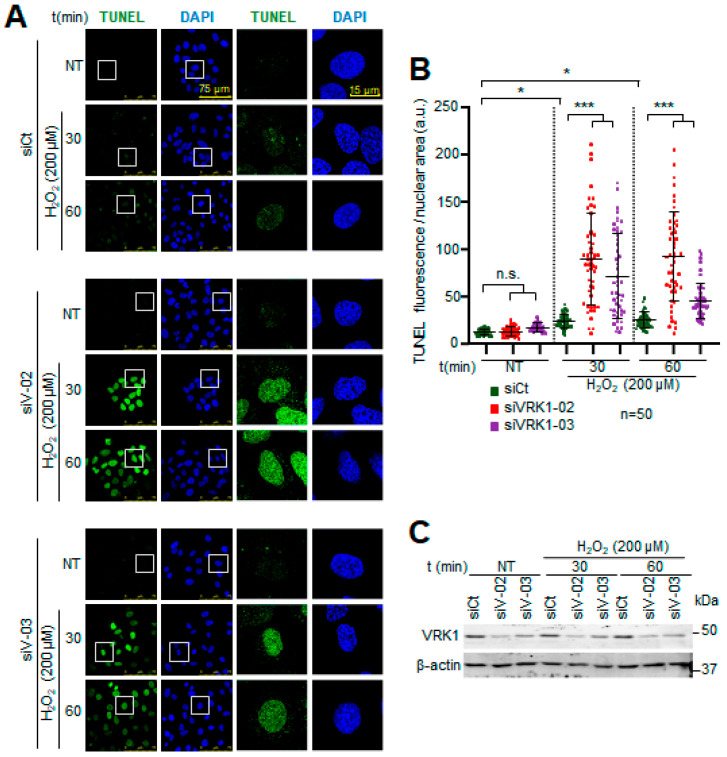
Accumulation of 3′-free DNA ends caused by hydrogen peroxide is enhanced by VRK1 depletion in A549 lung adenocarcinoma cells. (**A**) Image panels showing TUNEL levels stained by IF and detected using confocal microscopy. VRK1 was depleted using siVRK1-02 and si-VRK1-03 for 72 h. siControl was used as off-target siRNA control. Cells were treated with 200 µM H_2_O_2_ for 30 and 60 min. Squares on the left images are highlighting detailed cells shown on the right images of the panels. DAPI was used to stain nuclei. (**B**) Plot showing the quantification TUNEL levels of 50 cells for all conditions. (**C**) Immunoblot showing VRK1 levels. β-actin was used as a loading control. * *p* < 0.05, *** *p* < 0.001; n.s.: not significant. a.u.: arbitrary units; NT: non-treated; siCt: siControl; siV-02: siVRK1-02. siV-03: siVRK1-03. Experiments were independently performed three times.

The 8-oxoG DNA modifications caused by oxidative stress, if not repaired, can lead to DNA strand breaks, which can be detected by labeling the available free 3′-DNA ends with terminal deoxynucleotidyl transferase (TdT) in TUNEL assays. Hydrogen peroxide by itself causes low levels of single-strand DNA breaks, indicating that most of the 8-oxoG lesions were very efficiently repaired in the cells expressing VRK1 (Figure 2A, top panel). However, VRK1 depletion resulted in a significant accumulation of labeled free 3′-DNA ends (Figure 2A, center and bottom panels), indicating that altered chromatin as a consequence of VRK1 depletion impaired the repair of 8-oxoG lesions, which facilitated the progression towards ssDNA breaks in a short time after exposure to hydrogen peroxide. VRK1 depletion interferes with the detection and/or repair of these 8-oxoG DNA lesions caused by oxidative stress, which is consistent with the requirement of a specific nucleosomal organization for repair mediated by OGG1 [11,12,13]. This effect is a likely consequence of the alteration in the patterns of histone epigenetic modifications caused by VRK1 depletion [33,51]. The quantification is shown in Figure 2B. Altogether, these results suggest that VRK1 depletion, directly or indirectly, interferes with the oxidative stress repair mechanism and facilitates the accumulation of DNA strand breaks.

### 2.3. Oxidative Stress Induces Changes in the Epigenetic Modifications of H4K16, H3K9, and H3K4 That Are Impaired by VRK1 Depletion

**Figure 3:**
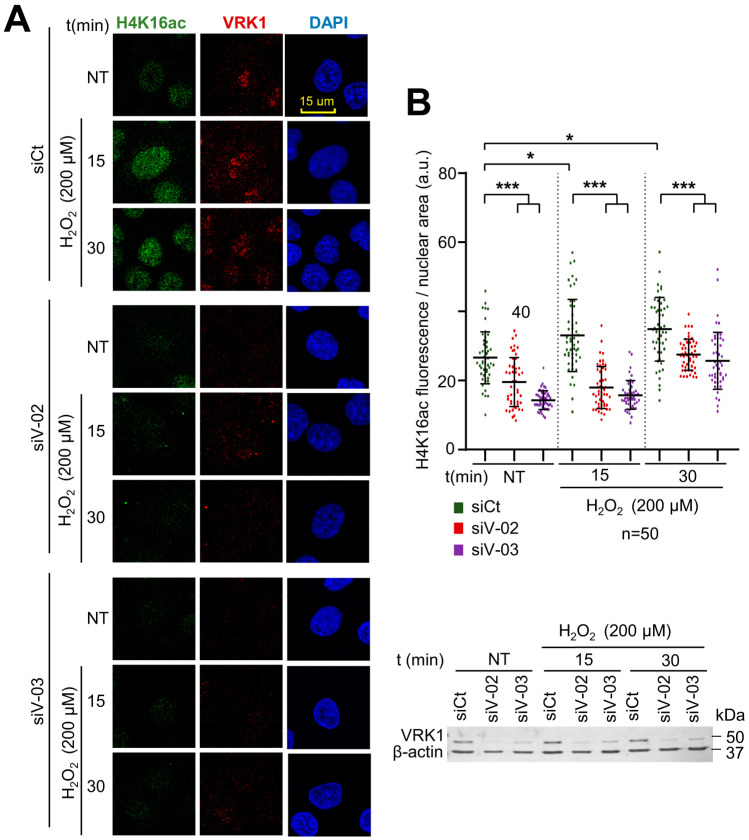
VRK1 depletion impairs the acetylation of H4K16 in A549 lung adenocarcinoma cells exposed to hydrogen peroxide. (**A**)**.** Image panels showing H4K16ac levels stained by IF and detected using confocal microscopy. DAPI was used to stain the nuclei and VRK1 for knockdown control. VRK1 was depleted using two siRNAs (siVRK1-02 and siVRK1-03) for 72 h. Cells were treated with 200 µM H_2_O_2_ for 15 and 30 min. (**B**) Quantification of H4K16ac fluorescence per nuclear area (a.u.: arbitrary units) of 50 cells (per condition) represented in a boxplot. Scale bar = 15 µm; * *p* < 0.05; *** *p* < 0.001; a.u.: arbitrary units; NT: non-treated; siCt: siControl; siV-02: siVRK1-02; siV-03: siVRK1-03. VRK1 levels were detected by immunoblot and are shown at the bottom. β-actin was used as a loading control. Experiments were independently performed three times.

**Figure 4:**
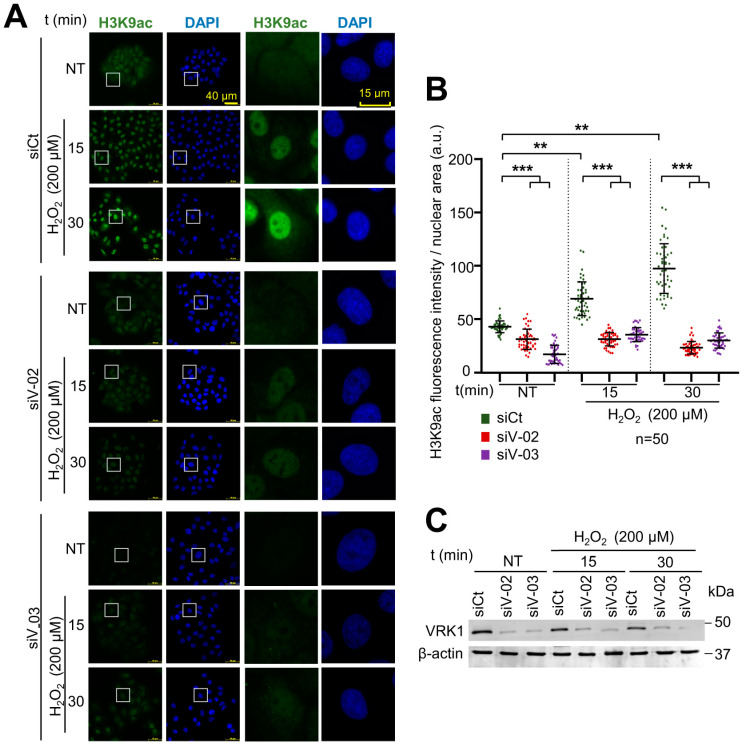
VRK1 depletion impairs the acetylation of H3K9 in A549 lung adenocarcinoma cells exposed to hydrogen peroxide. (**A**) Image panels showing H3K9ac levels stained by IF and detected using confocal microscopy. VRK1 was depleted using two siRNAs (siVRK1-02 and siVRK1-03) for 72 h. Cells were treated with 200 µM H_2_O_2_ for 15 and 30 min. (**B**) Quantification of H4K9ac fluorescence per nuclear area (a.u.) of 50 cells (per condition) represented in a boxplot. (**C**) VRK1 levels were detected by immunoblot and are shown at the bottom. β-actin was used as a loading control. Scale bar: 20 µm; ** *p* < 0.01, *** *p* < 0.001; NT: non-treated; a.u.: arbitrary units; siCt: siControl; siV-02: siVRK1-02; siV-03: siVRK1-03. Experiments were independently performed three times. Box: represent the detail.

**Figure 5:**
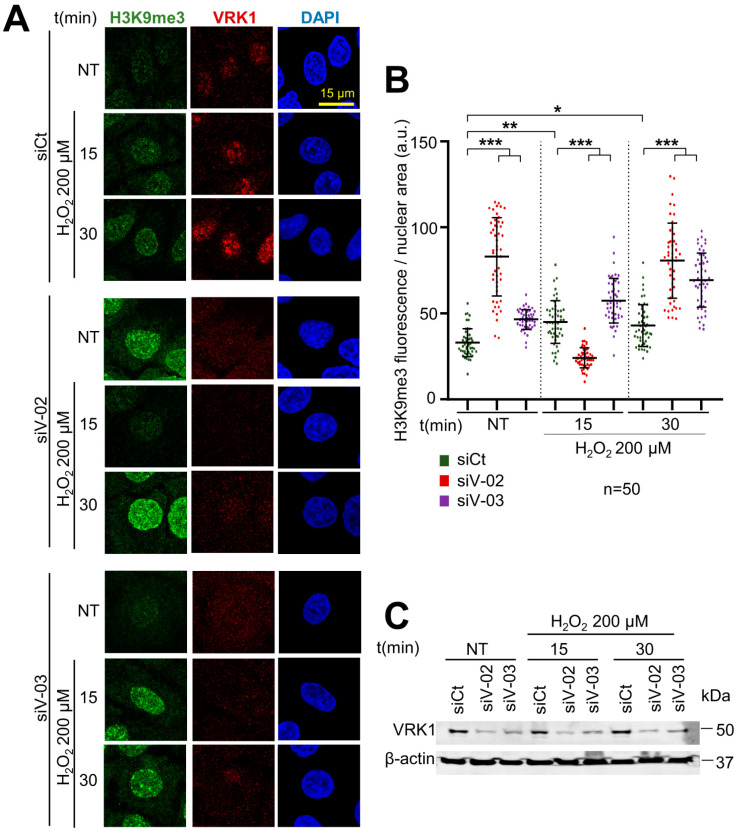
VRK1 depletion facilitates the trimethylation of H3K9 in A549 lung adenocarcinoma cells exposed to hydrogen peroxide. (**A**) Image panels showing trimethylation of H3K9 levels stained by IF and detected using confocal microscopy. VRK1 was depleted using two siRNAs (siVRK1-02 and siVRK1-03) for 72 h. Cells were treated with 200 µM H_2_O_2_ for 15 and 30 min. (**B**) Quantification of H3K9me3 fluorescence per nuclear area (a.u.) of 50 cells (per condition). (**C**) VRK1 levels were detected by immunoblot and are shown at the bottom. β-actin was used as a loading control. Scale bar = 15 µm; * *p* < 0.1; **; *p* < 0.01; *** *p* < 0.001; NT: non-treated; siCt: siControl; siV-02: siVRK1-02; siV-03: siVRK1-03. Experiments were independently performed three times.

**Figure 6:**
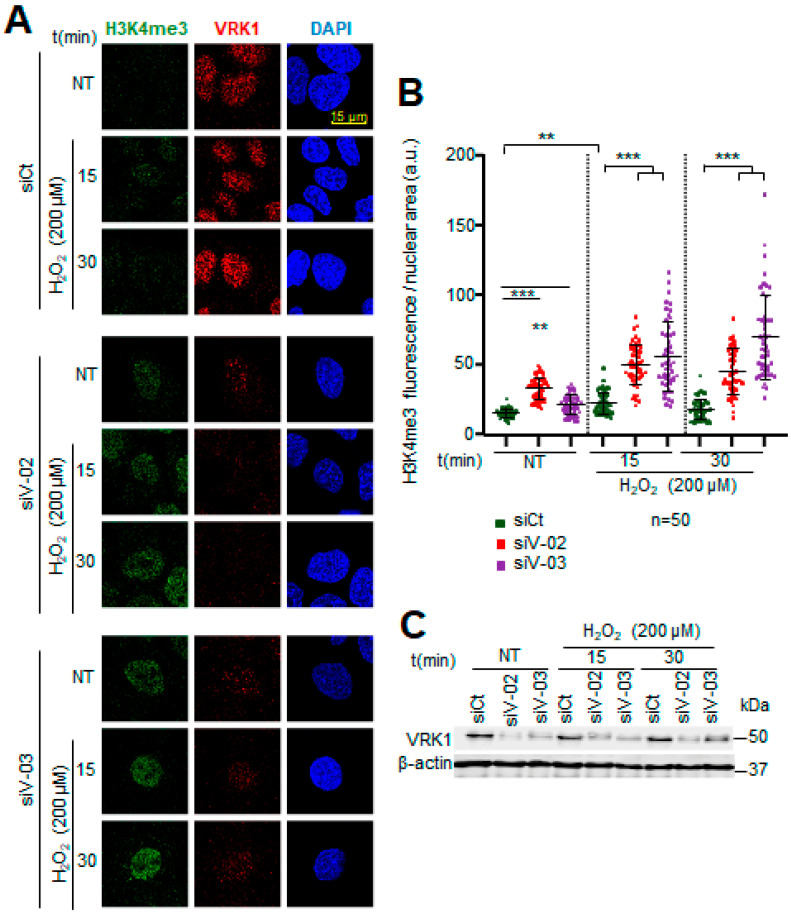
VRK1 depletion facilitates the trimethylation of H3K4 in A549 lung adenocarcinoma cells exposed to hydrogen peroxide. (**A**) Image panels showing trimethylation of H3K4 levels stained by IF and detected using confocal microscopy. VRK1 was depleted using two siRNAs (siVRK1-02 and siVRK1-03) for 72 h. Cells were treated with 200 µM H_2_O_2_ for 15 and 30 min. (**B**) Quantification of H3K4me3 fluorescence per nuclear area (a.u.: arbitrary units) of 50 cells (per condition). (**C**) VRK1 levels were detected by immunoblot and are shown at the bottom. β-actin was used as a loading control. Scale bar = 15 µm; ** *p* < 0.01; *** *p* < 0.001; NT: non-treated; siCt: siControl; siV-02: siVRK1-02; siV-03: siVRK1-03. Experiments were independently performed three times.

**Figure 7:**
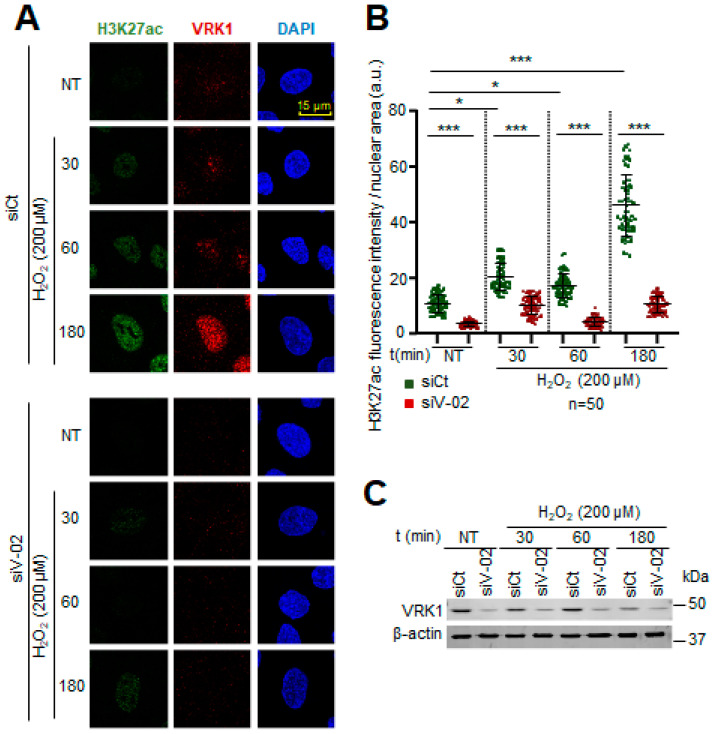
VRK1 depletion impairs the acetylation of H3K27 in A549 lung adenocarcinoma cells exposed to hydrogen peroxide. (**A**) Image panels showing H3K27ac levels stained by IF and detected using confocal microscopy. VRK1 was depleted using siVRK1-02 for 72 h. Cells were treated with 200 µM H_2_O_2_ for 15 and 30 min. (**B**) Quantification of H4K27ac fluorescence per nuclear area (a.u.) of 50 cells (per condition) represented by a dot blot. (**C**) VRK1 levels were detected by immunoblot and are shown at the bottom. β-actin was used as a loading control. Scale bar: 20 µm; * *p* < 0.1, *** *p* < 0.001; NT: non-treated; a.u.: arbitrary units; siCt: siControl; siV-02: siVRK1-02. Experiments were independently performed three times. Bars within a time point represent the comparison of siV-02 with respect to the siCt. Bars across different time points represent the comparison among the sCt values.

Histone post-translational modifications (PTMs) are crucial for chromatin remodeling and for many cellular processes, including the response to oxidative stress. To further analyze the role of VRK1 in chromatin remodeling, several histone epigenetic modifications, in particular, acetylations and methylations, which occur during the oxidative stress response, were analyzed in the presence and absence of VRK1 after exposure to hydrogen peroxide. A549 cells were treated with 200 µM H_2_O_2_ for 30, 60, and 180 min and were compared with non-treated cells. In these cells, the H4K16ac, H3K9ac, and H3K4me3 levels were detected using immunofluorescence techniques, which permits individual cell observation and confocal microscopy. The effects of oxidative stress were also determined in cells in which VRK1 was depleted using two different siRNAs (siVRK1-02 and siVRK1-03) for 72 h in A549 lung adenocarcinoma and LN229 glioblastoma cells. Oxidative stress induced an increase in the H4K16ac levels that were impaired by the VRK1 depletion in A549 cells (Figure 3) and in LN229 glioblastoma (Supplementary Figure S5). Oxidative stress caused an accumulation of H3K9ac levels, which was impaired by VRK1 depletion (Figure 4), and consequently led to an accumulation of the H3K9me3 levels in the cells treated with hydrogen peroxide and VRK1 depletion (Figure 5). The H3K4me3 levels increased in response to hydrogen peroxide treatment, which were enhanced by VRK1 depletion (Figure 6). Oxidative stress also caused an increase in the levels of H3K27ac, which were impaired by VRK1 depletion (Figure 7).

Based on these results, we can conclude that VRK1 alters different types of epigenetic histone post-translational modifications (PTM) and the nuclear phosphoproteome, and thus has a role in chromatin remodeling by regulating, directly or indirectly, and interfering with the response to oxidative stress. These findings indicate that VRK1 has an essential role in chromatin remodeling in the oxidative stress response since its loss completely alters the histone PTMs that are necessary for a proper DNA damage response.

### 2.4. VRK1 Depletion Alters the Nuclear Phosphoproteome of Chromatin-Associated Proteins in the Oxidative Stress Response

The defective response to oxidative DNA damage is likely to be a consequence of the effects that VRK1 can have on the chromatin organization and DNA damage response [60]. Therefore, oxidative damage and the effect of VRK1 are likely to alter, directly and indirectly, the covalent modifications of proteins in several nuclear signaling pathways. To detect the changes in phosphorylation, we performed a phosphoproteomic study of nuclear proteins in response to oxidative stress and the effect of VRK1 depletion on the phosphorylation of these proteins belonging to different nuclear signaling pathways.

**Figure 8:**
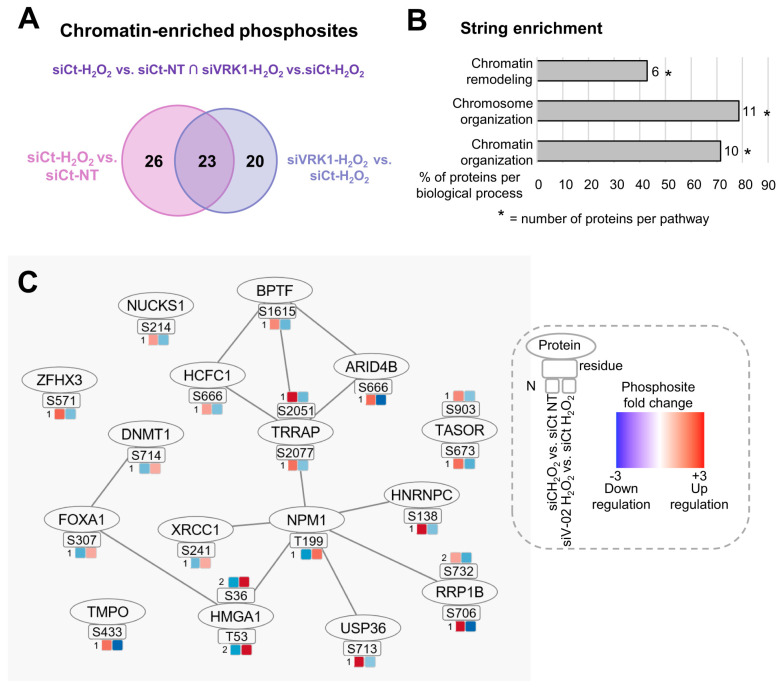
Overlapping significant chromatin-enriched phosphosites altered by both hydrogen peroxide treatment and VRK1 depletion in A549 lung adenocarcinoma cells. (**A**) Venn diagram representing the number of differentially phosphorylated phosphosites in each comparison and their intersection (used to build network in (**C**)). Numbers indicate unique or common genes (overlap) in each condition (circles). (**B**) Bar graph containing STRING enrichment of the proteins from B based on GO terms. (**C**) Protein–protein interaction (PPi) network showing overlapping significant phosphosites and proteins with fold changes (colored squares) of two group comparisons: H_2_O_2_-siControl vs. non-treated-siControl, and H_2_O_2_-siVRK1-02 vs. H_2_O_2-_siControl. N means the fold change. Nuclei isolation and knockdown controls are shown in Supplementary Figure S3. Heatmap containing the normalized phosphosite intensity of each replicate from each group is shown in Supplementary Figure S4. ∩ = intersection.

Because we observed that VRK1 activation takes place in the early response to oxidative stress, we first aimed to identify whether the VRK1 role in chromatin remodeling is associated with alterations in the nuclear phosphoproteome, necessary for a proper oxidative stress response. For this purpose, we performed quantitative phosphoproteomics analysis on A549 nuclear lysates from cells treated with hydrogen peroxide for 30 min in the presence and absence of VRK1 (Figure 8).

VRK1 was depleted in A549 cells using siVRK1-02 for 72 h, followed by treatment with 200 µM H_2_O_2_ for 30 min. TiOx phosphopeptide enrichment coupled to LC-MS/MS was performed to quantify the phosphoproteome changes in the nuclear fraction of A549 cells. Two independent biological replicates were measured in technical duplicates for each condition. First, we analyzed the significant events (−1.5 > FC > 1.5, *p* < 0.05) that overlapped in two two-group comparisons. The first comparison showed the effect of H_2_O_2_ on the phosphorylation of nuclear proteins compared to non-treated cells (non-treated-siCt versus H_2_O_2_-siCt). The second comparison indicated the effect of VRK1 knockdown in H_2_O_2_-treated cells on the phosphorylation of nuclear proteins compared to H_2_O_2_ siControl cells (H_2_O_2_-siCt versus H_2_O_2_-siVRK1) (Figure 8A). Then, we performed protein enrichment based on GO terms using String to filter the chromatin-related proteins. Oxidative damage induces the phosphorylation of the nuclear proteins associated with chromatin and chromosome organization, and several of them are affected by VRK1 depletion.

To characterize the specific processes in which these chromatin-related proteins are implicated, we performed Gene Ontology analysis using String enrichment on the proteins that composed the network. The String analysis results showed that 71% of the proteins were involved in the chromatin organization, 78% in the chromosome organization, and 42% in the chromatin remodeling (Figure 8B, Supplementary Table S1).

The proteins and phosphosites with significant fold changes from both comparisons are represented in Figure 8C, as well as protein–protein interactions built using String and Phosphopath (from Cytoscape) [61]. In the presence of H_2_O_2_ compared to non-treated cells, the phosphorylation of 75% of the phosphosites was upregulated and 25% downregulated. In the H_2_O_2_-siVRK1 cells compared to H_2_O_2_-siControl cells, the phosphorylation of 25% of the phosphosites was upregulated, and 75% was downregulated.

Based on these results, we can conclude that VRK1 alters different types of epigenetic enzymes that perform histone post-translational modifications (PTM) and the nuclear phosphoproteome and thus have a role in chromatin remodeling by regulating, directly or indirectly, the response to oxidative stress.

These findings indicate that VRK1 has an essential role in the chromatin remodeling in the oxidative stress response since its loss completely alters the histone PTMs that are necessary for a proper DNA damage response.

## 3. Discussion

Oxidative stress facilitates DNA strand breaks, causing a local alteration in chromatin [2], which leads to the activation of the VRK1 chromatin kinase, which is independent of the type of DNA damage [29,60] and triggers the appropriate DNA repair response, requiring the dynamic remodeling of the chromatin. VRK1 is a kinase that has an essential function in chromatin remodeling and in the response to DNA damage [53,62]. Moreover, the ROS levels, detected by the accumulation of 8-oxoG DNA lesions, increased in the cells lacking VRK1, indicating that, in the absence of this kinase, the regulation of ROS levels is disrupted, and alternative mechanisms can be implicated in the oxidative stress response. The increase in ROS levels in the absence of VRK1 can lead to different types of alterations in cellular and nuclear processes, affecting DNA and chromatin. Furthermore, VRK1 depletion by itself alters the pattern of epigenetic histone modifications [33], which can facilitate either the generation of DNA damage or impair its detection and repair.

Oxidative stress alters the phosphorylation pattern of the nuclear proteome. Many of these nuclear proteins are associated with chromatin roles, and their phosphorylation is impaired by VRK1 depletion. We have observed that treating cells with H_2_O_2_ caused the phosphorylation of several nuclear proteins, which was impaired when VRK1 was depleted, reverting to a state similar to the control. This analysis revealed that many chromatin-related proteins followed this pattern, such as DNMT1 (DNA methyl transferase 1), TASOR (Transcription activation suppressor), or TRRAP (Transformation/Transcription Domain Associated Protein), among others. Altogether, these results suggest that the chromatin organization under oxidative stress conditions may be regulated by VRK1, which alters the phosphorylation pattern of the essential nuclear and chromatin-related proteins that are implicated, directly or indirectly, in this response.

Epigenetic modifiers like the histone deacetylase HDAC1 are directly implicated in the repair of oxidative bases. The HDAC1 and HDAC2 activities decrease in the presence of oxidative stress [2]. HDAC1 activity is associated with an increase in 8-oxoG repair due to the deacetylation of the OGG1 promoter [63]. Additionally, the acetyltransferase Tip60/TRRAP complex promotes oxidative stress resistance by upregulating the expression of FOXO transcription factors through the acetylation of their promoter at H4K16 [64]. However, little is known about how epigenetic enzymes are controlled. Therefore, a better understanding of these enzymes is crucial to unveiling the mechanisms that may be involved in the dysregulation of the response to oxidative stress.

**Figure 9:**
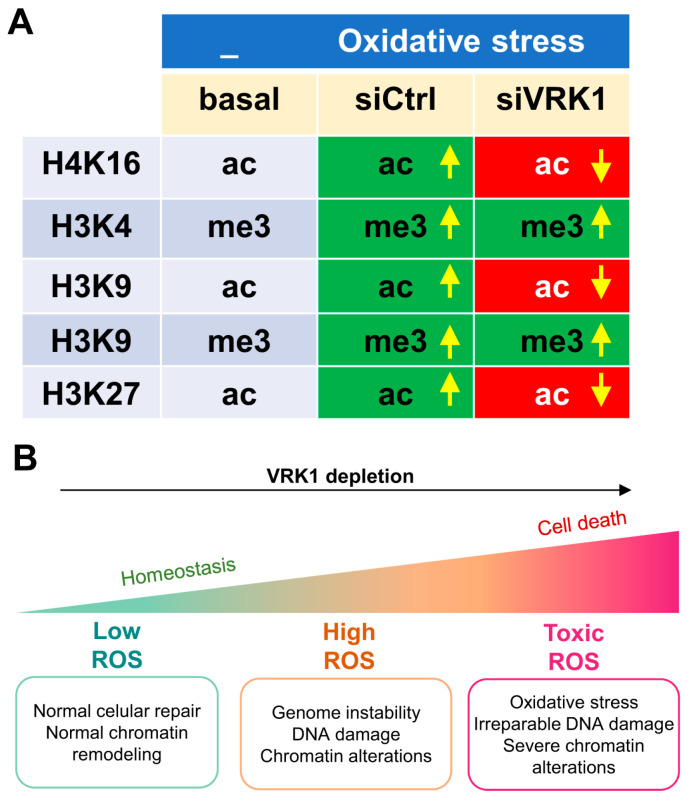
Pattern of H3 and H4 modifications altered by oxidative stress (**A**) and the effect of VRK1 depletion (**B**). Arrows indicate upregulation or down regulation. Each combination is likely to have a different pattern of protein interactions and of functional consequences in processes that require dynamic chromatin remodeling, which are associated with its organization and responses to DNA damage.

Histones H3 and H4 are histones modified by several types of epigenetic covalent modifications and have different combinations that can alter their recognition by other nuclear proteins and their functions. The increase in the methylation of H3K4 has been associated with elevated oxidative stress in cells [39]. In addition, changes in the acetylation of H3K9 and H4K16 have been detected in response to high ROS levels [2]. Upon demonstrating that VRK1 knockdown alters the phosphorylation pattern of the proteins that are implicated in chromatin remodeling in the oxidative stress response, we proposed that VRK1 depletion can also have an effect on the pattern of histone PTM alterations in the response to hydrogen peroxide exposure. In this work, we studied different histone epigenetic marks that are known to be altered in the presence of oxidative stress in cells. In particular, we studied H4K16ac, H3K9ac, and H3K4me3 as PTMs associated with open and relaxed chromatin conformations and H3K9me3, which is associated with compacted chromatin conformation. After hydrogen peroxide exposure, the above-mentioned open-chromatin PTM levels increased, and the closed chromatin mark levels decreased compared with non-treated cells (Figure 9). Moreover, in the VRK1-depleted cells, all the PTM patterns were reverted compared with the H_2_O_2_-treated siControl cells. These findings indicate that VRK1 is disrupting the chromatin organization in response to oxidative stress by altering the histone of the PTM landscape. It is interesting to note that residues H3K4 and H3K9 are next to Thr3 and Ser10, respectively, which are regulated by phosphorylation mediated by VRK1 [48], haspin [65], and AURKB [66]. In H3, the methylation of Lys4 impairs the phosphorylation of Thr3 and constitutes a phospho-methyl switch [67,68]. Thus, a negative charge in these phosphorylated residues, and their different combination in an individual histone, might influence the local pattern of acetylation and methylation, a process that will entail the recruitment of several nuclear enzyme activities, such as kinases, phosphatases, acetylases, deacetylases, methylases, and demethylases, and whose sequential contribution and coordination are not yet known and which might be different depending on the biological process. Therefore, deciphering and characterizing novel proteins that may contribute to this response are of utmost importance to comprehend the development of many human pathologies. Furthermore, the targeting of proteins that are involved in the oxidative stress response that is necessary for maintaining the non-lethal ROS levels in tumor cells may reveal novel strategies to induce tumor cell death due to ROS accumulation. The link between oxidative stress and chromatin remodeling has opened a door for new cancer therapies. Accumulating evidence supports the effectivity of targeting epigenetic enzymes as potential cancer treatments [1,69]. In this context, VRK1 depletion sensitizes cells to other therapeutic strategies based on DNA damage based on treatments using drugs such as olaparib [70], temozolomide [62], radiation, or doxorubicin [27]. Targeting VRK1 with a novel inhibitor that alters the histone pattern of histone epigenetic modifications [33,51] is a potential new strategy that requires development.

An additional implication regarding the role of VRK1 in the response to oxidative stress is its potential pathogenic role in severe neurological diseases. Very rare pathogenic VRK1 variants have been associated with distal neuropathies such as spinal muscular atrophy, amyotrophic lateral sclerosis, and hereditary spastic paraplegia [56], all of which have been linked to oxidative stress [71,72].

## 4. Material and Methods

### 4.1. Cell lines and Culture

Lung adenocarcinoma A549 (CCL-185) and glioblastoma LN229 (CRL-2611) cell lines were obtained from the ATCC and are mycoplasma-free. All cells were cultured as previously reported [62]. Cells were detached using trypsin-EDTA (TryplE™, Thermo Fisher Scientific, Waltham, MA, USA). Serum starvation (DMEM supplemented with 0.5% FBS, 50 U/mL penicillin, 50 µg/mL streptomycin, and 2 mM l-glutamine) was performed for 48 h when indicated. Cells were treated with different reagents, as specified in each section.

### 4.2. Kinase Assays

Cells were treated as indicated in the Section 2. Protein extracts (500 µg) were used for the immunoprecipitation of endogenous VRK1 [29]. The immunoprecipitated VRK1 was incubated with 1× kinase buffer (Supplementary Table S2), 5 µM ATP (Roche, Basel, Switzerland), and 5 µCi de [γ-^32^P] (PerkinElmer, Waltham, MA, USA) in agitation at 37 °C for 45 min. The radioactive signal was detected using Fuji Medical X-ray films.

### 4.3. Interference RNA (siRNA) Transfection

Two siRNA, si-VRK1-02 (5′-CAAGGAACCUGGUGUUGAA-3′) and si-VRK1-03 (5′-GGAAUGGAAAGUAGGAUUA-3′), were used to deplete VRK1 as previously reported [62]. ON-TARGET plus siControl (siCt) non-targeting siRNA (5′-UGGUUUACAUGUCGACUAA-3′) was used as a negative control [62]. All siRNAs were from Dharmacon RNA Technologies, Lafayette, CO, USA. Opti-MEM was used for lipotransfectine (Solmeglas, Madrid, Spain) and siRNA dilution in Opti-MEM (GIBCO-Life Technologies, Carlsbad, CA, USA) according to the manufacturer guidelines. siRNAs were diluted in Opti-MEM, used at 200 nM, and added to the lipotransfectin-Opti-MEM mix. The lipotransfectine-Opti-MEM-siRNA mix was incubated for 30 min and added gently to cells, which were maintained in antibiotic-free media.

### 4.4. Immunofluorescence and Microscopy

Immunofluorescence (IF) assays were performed as previously reported [29,62]. The primary antibodies used are shown in Supplementary Table S3. Cell lines were cultured on glass coverslips (Thermo Fisher Scientific) in culture dishes. At the indicated times, cells were fixed with a 3% paraformaldehyde (PFA) solution in PBS for 20 min at RT, removed, and 200 mM glycine was added to reduce the excess of aldehyde groups, followed by cell permeabilization with 0.2% Triton X-100 for 20 min. Later, cells were blocked with 1% BSA diluted in PBS containing 0.1% sodium azide for 1 h at room temperature (RT), or overnight (o/n) at 4 °C. The first primary antibody was incubated from 2 to 4 h at RT or o/n at 4 °C. Coverslips were washed 3 times with PBS and the second primary antibody (if necessary) was incubated between 2 and 4 h at RT. Afterward, cells were washed with PBS and incubated with the secondary antibodies (Supplementary Table S4) at 1:1000 dilution for 1 h at RT. Cells were washed 3 times in PBS. DAPI (4′, 6-diamidino-2-phenylindole) was used to stain nuclei at 1:1000 dilution for 15 min. Coverslips were washed 3 times with PBS and mounted into microscope slides with MOWIOL 4-88 (Calbiochem, San Diego, CA, USA). The experiments were independently performed three times.

Immunofluorescence images were obtained using a Leica TCS SP5 inverted fluorescence confocal microscope (Leica Microsystems, Wetzlar, Germany) connected to a Leica DC100 (Leica Microsystems) digital video camera that was used to take images. Image analysis was conducted using ImageJ software (NIH, version 1.53t).

### 4.5. Immunofluorescence Staining for 8-oxoG Determination

Cells were fixed in 4% PFA for 15 min. Then, they were washed 3 times with PBS and permeabilized with 0.2% Triton X-100 for 10 min. Cells were incubated with 4.5 N HCl. After that, cells were blocked in 4% BSA, 0.5% Triton X-100 in PBS, and incubated with 8-oxoG primary antibody (1:400) overnight at 4 °C. The experiments were independently performed three times.

### 4.6. TUNEL Assays

Cells were fixed, permeabilized, and blocked as described above. Free 3′-OH DNA ends were labeled with Fluorescein-dUTP by terminal deoxynucleotidyl transferase and detected according to the manufacturer guidelines. TUNEL assay in situ detection (Roche). The experiments were independently performed three times.

### 4.7. Protein Extraction and Quantification

All steps of protein extraction were carried out on ice as reported [29,62]. Briefly, cells were lysed in lysis buffer (Supplementary Table S2) supplemented with protease inhibitors (1 mM PMSF, 10 µg/mL aprotinin, and 10 µg/mL leupeptin) and phosphatase inhibitors (1 mM sodium orthovanadate and 1 mM sodium fluoride). Lysates were incubated for 20 min on ice and then centrifuged at 13,000× *g* for 20 min. Pellet was discarded. Soluble fractions were kept and stored at −20 °C. Protein concentration was determined using the BCA protein assay kit (Thermo Fisher Scientific).

### 4.8. SDS-PAGE Electrophoresis and Immunoblots

The running gel is composed of 7.5–12.5% acrylamide, 0.13–0.4% bis-acrylamide in 0.375 M Tris-HCl (pH 8.8) and 3.5 mM SDS, tetramethylethylenediamine (TEMED), and ammonium persulfate (APS). Three different acrylamide–bisacrylamide percentages for running gels were used varying according to the size of the target protein. Further, 12.5–0.33% gels were used for small proteins (<30 kDa) and 10–0.27% gels for proteins between 30 and 100 kDa. The stacking gel contained 4.8% acrylamide, 0.128% bisacrylamide in 0.125 M Tris-HCl (pH 6.8), and 3.5 mM SDS (sodium dodecyl sulfate). Electrophoresis was carried out under denaturing conditions in electrophoresis buffer as reported [30]. Precision Plus Protein Standards Dual Color (Bio-Rad, Shinagawa City, Tokyo) was used as a molecular weight protein reference. Sodium dodecyl sulfate polyacrylamide gel electrophoresis (SDS-PAGE) was performed under a constant voltage (90 V for 15 min and 120 V for 80 min).

After SDS-PAGE electrophoresis, proteins were transferred to PVDF Immobilon-P/FL/P^Sq^ membranes (Millipore, Burlington, MA, USA), which were activated in methanol (Sigma Aldrich, St. Louis, MO, USA) for 2 min. The transfer was performed at 90 V for 90 min at 4 °C, followed by blocking with 5% non-fat dried milk or BSA, diluted in TBS-T for 1 h at RT. Afterward, membranes were washed 3 times in TBS-T and incubated with the primary antibody in 1% BSA in TBS-T and diluted according to the manufacturer specifications for 1–2 h at RT or overnight at 4 °C. Membranes were washed 3 times with TBS-T and incubated with the secondary antibody (Table S4 at 1:10,000 dilution in 1% BSA in TBS-T in the dark. Membranes were washed 3 times in TBS-T and scanned in the Odyssey Infrared Imaging System (LI-COR Biosciences, Lincoln, NE, USA). Bands in membrane images were quantified using Quantity One software version 29.

### 4.9. Statistics and Data Analysis

Statistics were performed with SPSS versions 25 and 26 and GraphPad Prism version 8.0.1. Kruskal–Wallis statistical test for non-parametric distributions was used for two-group comparisons in all experiments except for phosphoproteomics experiments. Differences between groups were considered significant when *p*-values < 0.01.

### 4.10. Cytoplasm/Nucleus Fractionation for Phosphoproteomics Analysis

First, cells treated as specified in the Section 2 were lysed in cytosolic lysis buffer with phosphatase (PhoSTOP from Sigma-Aldrich) and protease inhibitors (cOmplete from Sigma-Aldrich) were added right before use, incubated on ice for 5 min, and centrifuged at 2000× *g* for 5 min at 4 °C. The cytosolic fraction (supernatant) was flash-frozen in liquid nitrogen and stored at −80 °C. The pellet (nuclear fraction) was washed 3 times in cytosolic lysis buffer without IGEPAL. Nuclei pellets were lysed in nuclear lysis buffer with phosphatase and protease inhibitors added right before use. Nuclear lysates were incubated in ice for 10 min and sonicated in 2 cycles of 15 s (21% amplitude) with a digital sonifier-SFX250 (Branson Ultrasonic, Brookfield, CT, USA). Then, nuclear lysates were centrifuged at 16,000× *g* for 10 min at 4 °C and the supernatants containing nuclear proteins were flash-frozen in liquid nitrogen and stored at −80 °C. BCA protein assay kit (Thermo Fisher Scientific) was used to determine protein concentration.

### 4.11. Sample Preparation for Phosphoproteomics Analysis

Nuclear lysates were reduced with 10 mM dithiothreitol (DTT) for 45 min at 56 °C and alkylated with 50 mM iodoacetamide (IAA) for 45 min at RT in the dark. Lysates were digested with trypsin in a proportion of 20 µg trypsin/mg protein o/n at RT. Further, 0.1% trifluoroacetic acid (TFA) was added to the peptides. Peptides were desalted using OASIS HLB Cartridge commercial kit in 10 mg columns following the manufacturer’s protocol. Phospho-enrichment was performed using immobilized metal affinity chromatography (IMAC) automatically using high-capacity Agilent AssayMAP Fe (III)-NTA cartridges (Santa Clara, CA, USA) in the AssayMAP Bravo as previously described^127^. Peptides were lyophilized for 45 min at 45 °C in a vacuum centrifuge.

### 4.12. Label-Free Phosphoproteomics by Liquid Chromatography Tandem Mass Spectrometry (LC)-ms/ms

Nuclear lysates and phosphopeptide-enriched samples were diluted in 4% acetonitrile (ACN) and 0.5% TFA before use. Peptides were separated on a Reprosil C18 aqua column (1.9 µm porous spherical silica, 120 Å pore diameter) in a 90-min buffer gradient (2–32% ACN, 0.5% acetic acid) at a rate of 300 nL/min using a U3000 RSLC high-pressure nano LC (Dionex LC-Packings)(Thermo-Fisher Scientific, Waltham, MA, USA). After injection, peptides were measured online and nanospray ionization was performed using a Q Exactive mass spectrometer (Thermo Fisher Scientific) [73].

### 4.13. Protein, Phosphopeptide and Phosphorylation Site Identification

MaxQuant v2.0.3.0 [74] was used for MS/MS spectra search against the Uniprot human reference proteome FASTA from 2021 containing 42,383 entries [75] according to the previously defined settings [73]. Trypsin was selected as the cutting enzyme and two missing cleavages were permitted. Ser, Thr, and Tyr phosphorylation (+79.966330 Da) were treated as variable modifications. Peptide precursor ions and fragment ions were searched with a maximum and minimum mass deviation of 4.5 ppm and 20 ppm, respectively. Protein, peptide, and phosphosite identifications were filtered at an FDR of 1%. The minimum Andromeda score for modified peptides was set as 40 (delta score 17). The minimum peptide length allowed was 7 amino acids. Match-between-runs and label-free quantification settings were selected.

### 4.14. Phosphoproteomics Data Analysis

Normalization, clustering, and statistical tests were performed using RStudio v4.1.3. Phosphopeptides were quantified by phosphorylation intensities. Phosphorylation intensities were normalized to the median intensity of all phosphopeptides identified in the sample. Phosphosites with a localization probability > 0.75 (class I phosphosites) were used in further analyses. Multiplicity of 1, 2, or 3 was designated to each phosphosite to categorize phosphosites identified in mono-phosphorylated, diphosphorylated, or >diphosphorylated peptides, respectively. Phosphosite fold change (FC) and *p*-value between group comparisons were calculated using Limma package from RStudio [76]. The mass spectrometry proteomics data have been deposited to the ProteomeXchange Consortium via the PRIDE partner repository with the dataset identifier PXD040944 [77,78].

In two-group comparisons, phosphosite intensities with a FC > 1.5 or <−1.5 and a *p*-value < 0.05 were considered significantly different. Missing values were excluded from statistical analysis. Two biological replicates in technical duplicates were measured for each condition. Pearson correlation between replicates was excellent.

Post-translational Modification Signature Enrichment Analysis (PTMsea) was used for PTM enrichment. PTMsea is based on a PTM signatures database (PTMsigDB) that provides curated phosphorylated signatures of kinases, perturbations, and signaling pathways to enable site-specific PTM signature enrichment analysis [79]. PTMsea analysis was used as a GenePattern module. Phosphosite-specific pathway enrichment of two-group comparisons was analyzed using this tool.

Phosphopath from Cytoscape was used to build protein–protein interaction (PPi) networks representing detected phosphosites of each protein [80]. Each phosphosite was represented together with the fold change of 2-group comparisons. STRING was used to perform protein enrichment and filter proteins based on their functions as indicated in the Section 2 [81].

## 5. Conclusions

Our findings indicate that VRK1 has a role in the early oxidative stress response by altering the nuclear phosphoproteome and the pattern of epigenetic modifications and, consequently, changing the histone PTM landscape, jeopardizing chromatin remodeling and sensitizing cells to DNA damage by oxidative stress as a result of the reduced levels of VRK1. This implication could favor understanding the underlying pathological mechanisms due to a defective oxidative stress response that leads to human diseases like cancer. Alternatively, VRK1 inhibition could emerge as a novel therapeutic target because it may facilitate the accumulation of toxic ROS in tumor cells in combination with other cancer drugs through the impairment of chromatin remodeling, resulting in decreased tumor cell viability and death. Therefore, targeting VRK1 in combination with histone epigenetic inhibitors can lead to novel synthetic lethality strategies in cancer treatment [82].

## Data Availability

The mass spectrometry proteomics data are freely available and have been uploaded to ProteomeXchange [78] via the PRIDE partner repository [77] with the dataset identifier PXD040944.

## Supplementary Materials

The supporting information can be downloaded at https://www.mdpi.com/article/10.3390/ijms25094874/s1. Reference [83] is cited in Supplementary Materials.
