# Supplementary material for: VRK1 Regulates Sensitivity to Oxidative Stress by Altering Histone Epigenetic Modifications and the Nuclear Phosphoproteome in Tumor Cells
Source: Int J Mol Sci. 2024 Apr 30;25(9):4874. doi: 10.3390/ijms25094874 (PMC11084957; doi:10.3390/ijms25094874)
Supplement: Supplementary file 1 [file ijms-25-04874-s001.zip › Figure S1.pdf]

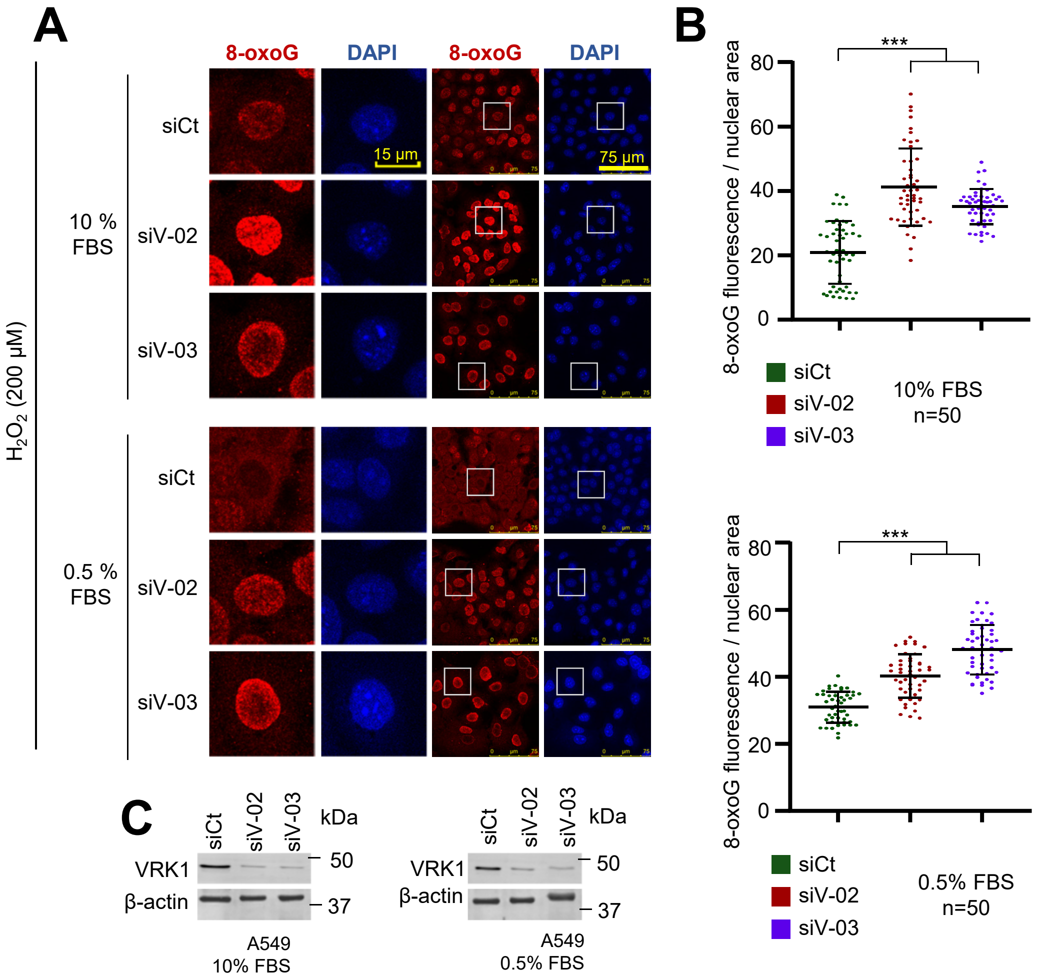

Figure S1. The accumulation of 8-oxoG DNA lesions induced by hydrogen perooxide in A549 lung adenocarcinoma cells is independent of the presence of serum. **A**. Immunofluorescene of A549 cells treated with hydrogen peroxide in the presence of serum (top) or absence (bottom) after depetion of VRK1. The boxes indicate the individual cell shown in the left side panel. . **B.** Quantification of the levels of 8-oxoG shown in part A. **C.** immunoblot showing the depletion of VRK1. *** $p < 0.001$. siCt: control; siV-02: siVRK1.02; siV-03: siVRK1-03. NT: non treated. The experiment was independently performed three times.
