# Supplementary material for: VRK1 Regulates Sensitivity to Oxidative Stress by Altering Histone Epigenetic Modifications and the Nuclear Phosphoproteome in Tumor Cells
Source: Int J Mol Sci. 2024 Apr 30;25(9):4874. doi: 10.3390/ijms25094874 (PMC11084957; doi:10.3390/ijms25094874)
Supplement: Supplementary file 1 [file ijms-25-04874-s001.zip › Figure S2.pdf]

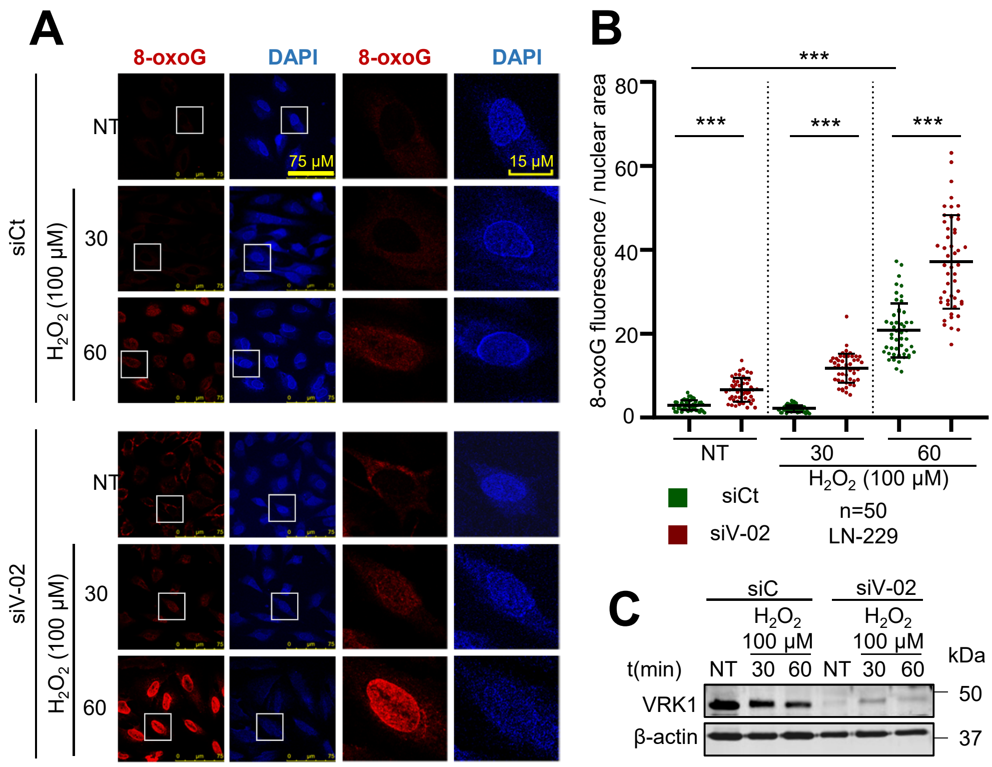

Figure S2. Accumulation of 8-oxoG DNA lesions induced by hydrogen peroxide in LN-229 glioblastoma cells that were serum deprived. **A**. Immunofluorescence of LN-229 cells treated with hydrogen peroxide in the absence after depletion of VRK1. The boxes indicate the individual cell shown in the right side panel. **B.** Quantification of the levels of 8-oxoG shown in part A. **C.** Immunoblot showing the depletion of VRK1. *** $p < 0.001$. siCt: control; siV-02: siVRK1.02; NT: non treated. The experiment was independently performed three times.
