# Supplementary material for: VRK1 Regulates Sensitivity to Oxidative Stress by Altering Histone Epigenetic Modifications and the Nuclear Phosphoproteome in Tumor Cells
Source: Int J Mol Sci. 2024 Apr 30;25(9):4874. doi: 10.3390/ijms25094874 (PMC11084957; doi:10.3390/ijms25094874)
Supplement: Supplementary file 1 [file ijms-25-04874-s001.zip › Figure S3.pdf]

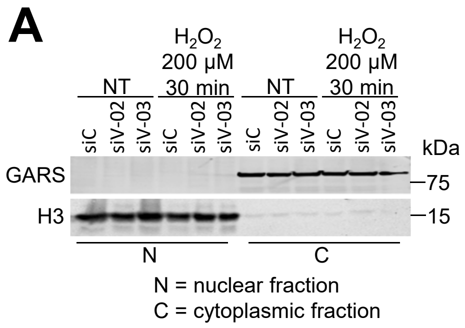

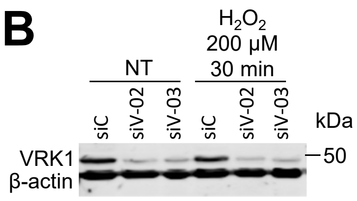

C

siC NT replicate correlation	siC NT - B1 T1	siC NT - B1 T2	siC NT - B2 T1	siC NT - B2 T2
siC NT - B1 T1	1	0.921029373	0.867927977	0.838593824
siC NT - B1 T2	0.921029373	1	0.856512986	0.868671301
siC NT - B2 T1	0.867927977	0.856512986	1	0.980188376
siC NT - B2 T2	0.838593824	0.868671301	0.980188376	1

si02 NT replicate correlation	si02 NT - B1 T1	si02 NT - B1 T2	si02 NT - B2 T1	si02 NT - B2 T2
si02 NT - B1 T1	1	0.974218979	0.97976631	0.971521117
si02 NT - B1 T2	0.974218979	1	0.976591914	0.983902432
si02 NT - B2 T1	0.97976631	0.976591914	1	0.990871891
si02 NT - B2 T2	0.971521117	0.983902432	0.990871891	1

siC H_2O_2 replicate correlation	siC H_2O_2 - B1 T1	siC H_2O_2 - B1 T2	siC H_2O_2 - B2 T1	siC H_2O_2 - B2 T2
siC H_2O_2 - B1 T1	1	0.969695564	0.967058833	0.956873882
siC H_2O_2 - B1 T2	0.969695564	1	0.97135893	0.977741335
siC H_2O_2 - B2 T1	0.967058833	0.97135893	1	0.989375949
siC H_2O_2 - B2 T2	0.956873882	0.977741335	0.989375949	1

si02 H_2O_2 replicate correlation	si02 H_2O_2 - B1 T1	si02 H_2O_2 - B1 T2	si02 H_2O_2 - B2 T1	si02 H_2O_2 - B2 T2
si02 H_2O_2 - B1 T1	1	0.976995405	0.978361789	0.96898722
si02 H_2O_2 - B1 T2	0.976995405	1	0.98039933	0.983442206
si02 H_2O_2 - B2 T1	0.978361789	0.98039933	1	0.992008872
si02 H_2O_2 - B2 T2	0.96898722	0.983442206	0.992008872	1

Figure S3. Nuclei isolation and replica correlations. **A**. Fractionation of nuclear (histone H3 as marker) and cytoplasmic proteins (GARS as marker). **B**. Control showing in immunoblots the effect of VRK1 depletion on its levels. **C**. Correlation among the replicas of the experiments.
