# Supplementary material for: VRK1 Regulates Sensitivity to Oxidative Stress by Altering Histone Epigenetic Modifications and the Nuclear Phosphoproteome in Tumor Cells
Source: Int J Mol Sci. 2024 Apr 30;25(9):4874. doi: 10.3390/ijms25094874 (PMC11084957; doi:10.3390/ijms25094874)
Supplement: Supplementary file 1 [file ijms-25-04874-s001.zip › Figure S4.pdf]

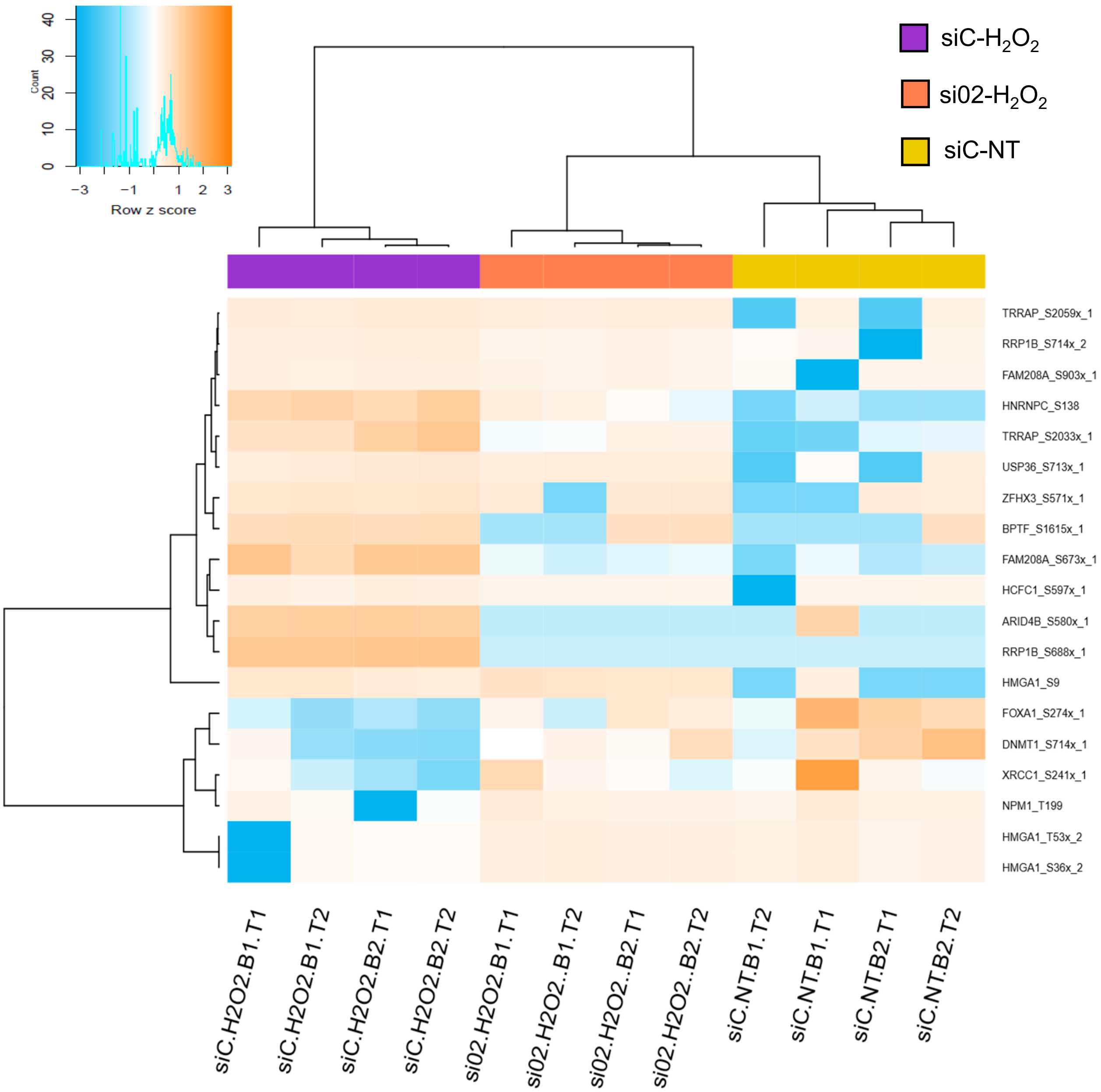

Figure S4. Phosphorylation intensities of phosphoproteomics replicates in chromatin-related H2O2-treated A549 cells Heatmap showing phosphorylation intensities of the overlapping phosphosites differentially expressed in chromatin-enriched proteins after VRK1 knock-down and 30 min of 200 μM H_2O_2 treatment. Row z score of two biological replicates in technical duplicates is shown. siC = siControl; si02 = siVRK1-02; NT = non-treated; H_2O_2 = H_2O_2 200 μM 30 min; B1 = biological replicate 1; B2 = biological replicate 2; T1 = technical replicate 1; T2 = technical replicate 2.
