# Supplementary material for: VRK1 Regulates Sensitivity to Oxidative Stress by Altering Histone Epigenetic Modifications and the Nuclear Phosphoproteome in Tumor Cells
Source: Int J Mol Sci. 2024 Apr 30;25(9):4874. doi: 10.3390/ijms25094874 (PMC11084957; doi:10.3390/ijms25094874)
Supplement: Supplementary file 1 [file ijms-25-04874-s001.zip › Figure S5.pdf]

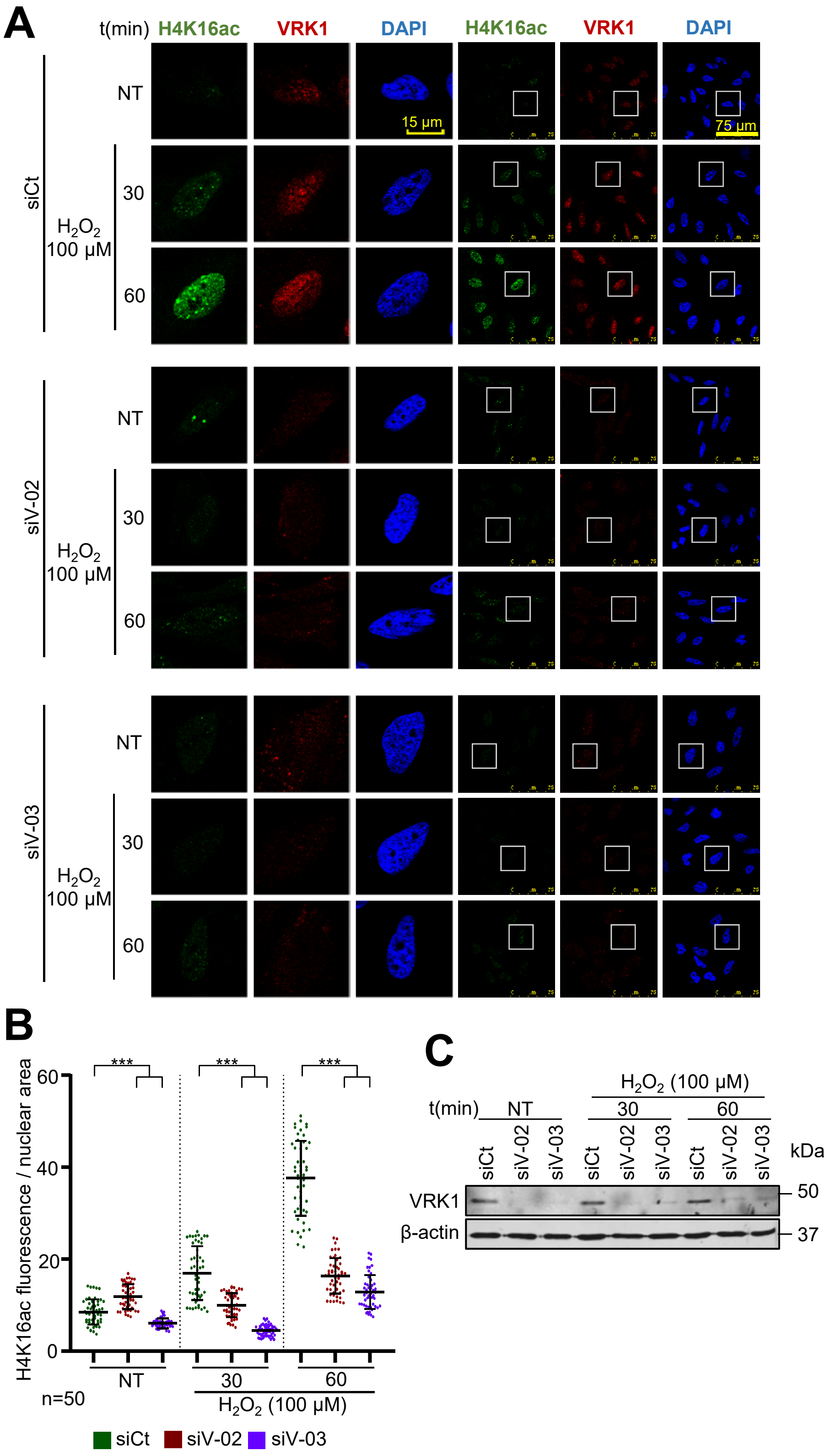

Figure S5. VRK1 depletion induces the decrease of H4K16ac after treatment with hydrogen peroxide in LN-229 glioblastoma cells. **A**. Immnunofluorescene. The boxes indicate the individual cells shown. **B.** Quantification of the level of H4K16 acetylation. **C**. Immunoblot showing the depletion of VRK1. *** $p < 0.001$. NT: not treated. The experiment was independently performed three times.
