# Supplementary material for: VRK1 Regulates Sensitivity to Oxidative Stress by Altering Histone Epigenetic Modifications and the Nuclear Phosphoproteome in Tumor Cells
Source: Int J Mol Sci. 2024 Apr 30;25(9):4874. doi: 10.3390/ijms25094874 (PMC11084957; doi:10.3390/ijms25094874)
Supplement: Supplementary file 1 [file ijms-25-04874-s001.zip › Supplementary Table S2.pdf]

Supplementary Table S2. Composition of buffers.

Buffers	Composition
**Lysis**	50 mM Tris-HCl (pH 8.0), 150 mM NaCl, 1% Triton X-100 and 1 mM EDTA.
**Kinase assay**	20mM Tris-HCl (pH.5), 5mM $MgCL_2$, 0.5 mM DTT, 150 mM KCl, 5 μM ATP and 5 μCi ATP-[γ-^{32}P]
**Sample**	62.5 mM Tris-HCl (pH 6.8), 10% glycerol, 2.3% SDS, 0.1% bromophenol blue, and 5% β-mercaptoethanol
**Electrophoresis**	25 mM Tris-HCl, 200 mM glycine and 1.7 mM SDS
**Transfer**	25 mM Tris-HCl, 19.2 mM glycine and 10-20% methanol
**TBS-T**	25 mM Tris-HCl (pH 8.0), 50 mM NaCl, 2.5 mM KCl and 0.1% Tween-20
**Cytosolic**	10 mM HEPES pH 7.5, 40 mM KCl, 3 mM $MgCl_{2,}$ 5% glycerol, 2.5% IGEPAL
**Nuclear**	20 mM HEPES pH 8, 9 M urea
