# Supplementary material for: VRK1 Regulates Sensitivity to Oxidative Stress by Altering Histone Epigenetic Modifications and the Nuclear Phosphoproteome in Tumor Cells
Source: Int J Mol Sci. 2024 Apr 30;25(9):4874. doi: 10.3390/ijms25094874 (PMC11084957; doi:10.3390/ijms25094874)
Supplement: Supplementary file 1 [file ijms-25-04874-s001.zip › Supplementary Table S3.pdf]

Supplementary Table S3. Primary antibodies.

Target	antibody	Dilution (WB/IF)	Clone, reference	Supplier
VRK1	Mouse monoclonal	- / 1:1000	1B5	[1]
VRK1	Mouse monoclonal	1:1000 / -	1F6	[1]
VRK1	Rabbit polyclonal	1:1000 / -	VC	[1]
β-actin	Mouse monoclonal	1:2000 / -	AC15 A5441	Sigma-Aldrich
H3K4me3	Rabbit polyclonal	- /1:1000	9727	Cell signaling
H3K9ac	Rabbit polyclonal	- / 1:1000	07-352	Millipore
H3K9me3	Rabbit polyclonal	- /1:800	07-442	Millipore
H3K27ac	Rabbit polyclonal	- /1:1000	07-360	Millipore
Histone H3	Rabbit polyclonal	1:1000 / -	9715	Cell signaling
H4K16ac	Rabbit monoclonal	- / 1:1000	Ab109463	Abcam
8-oxoGuanine (8-oxoG)	Mouse monoclonal	- / 1:500	483.15 MAB3560	Millipore
GARS	Mouse monoclonal	- / 1:1000	VMA00198	Bio Rad

1. Valbuena A, Lopez-Sanchez I, Vega FM, Sevilla A, Sanz-Garcia M, Blanco S, Lazo PA: **Identification of a dominant epitope in human vaccinia-related kinase 1 (VRK1) and detection of different intracellular subpopulations**. *Arch Biochem Biophys* 2007, **465**(1):219-226. Doi 10.1016/j.abb.2007.06.005
