# Supplementary material for: VRK1 Regulates Sensitivity to Oxidative Stress by Altering Histone Epigenetic Modifications and the Nuclear Phosphoproteome in Tumor Cells
Source: Int J Mol Sci. 2024 Apr 30;25(9):4874. doi: 10.3390/ijms25094874 (PMC11084957; doi:10.3390/ijms25094874)
Supplement: Supplementary file 1 [file ijms-25-04874-s001.zip › Supplementary Table S4.pdf]

Supplementary Table S4. Secondary antibodies.

Antibody	Fluorochrome	Use (dilution)	Reference, Supplier
Goat anti-Mouse IgG	Cy3 (Red)	IF (1:1000)	115-165-146, Jackson ImmunoResearch
Goat anti-Rabbit IgG	Cy2 (Green)	IF (1:1000)	111-225-144, Jackson ImmunoResearch
Goat anti-Mouse IgG	DyLight 680 (Red)	WB (1:10000)	35518, Thermo Fisher Scientific
Goat anti-Rabbit IgG	DyLight 800 (Green)	WB (1:10000)	35571, Thermo Fisher Scientific
